# Sump syndrome: Diagnosis dilemmas and therapeutic approaches—A case series

**DOI:** 10.1002/ccr3.9378

**Published:** 2024-10-31

**Authors:** Shahem Abbarh, Bisher Sawaf, Hussam Almasri, Girisha Balaraju, Mhd Kutaiba Albuni, Shaher Abbarah, Ashraf I. Ahmed, Abdellatif Ismail, Saad Rashid Mohammad Al Kaabi

**Affiliations:** ^1^ Department of Internal Medicine Hamad Medical Corporation Doha Qatar; ^2^ Department of Internal Medicine University of North Dakota Fargo North Dakota USA; ^3^ Department of Internal Medicine Trihealth Good Samaritan Hospital Cincinnati Ohio USA; ^4^ Department of General Surgery College of Medicine, Almaarefa University Riyadh Saudi Arabia; ^5^ College of Medicine, QU Health, Qatar University Doha Qatar; ^6^ Department of Internal Medicine University of Maryland Medical Center‐Midtown Campus Baltimore Maryland USA

**Keywords:** cholangitis, choledochoduodenostomy (CDD), endoscopic retrograde cholangiopancreatography (ERCP), pneumobilia, sump syndrome

## Abstract

**Key Clinical Message:**

It is important to consider the diagnosis of Sump syndrome in patients with a history of open cholecystectomy, particularly in those who migrate from developing countries where alternative biliary interventions may be limited. The presentation may range from acute severe, mimicking acute ascending cholangitis, to chronic recurrent abdominal pain without evidence of inflammation. Management is a case‐by‐case decision, with principal management aims to decompress the biliary tract and address any underlying sepsis.

**Abstract:**

Sump syndrome is a rare and often long‐term complication of choledochoduodenostomy (CDD). The presentation and severity are variable, and management should be tailored to each patient based on several factors. Herein, we report three cases of sump syndrome, each demonstrating unique diagnostic dilemmas and therapeutic modalities. Case I describes a woman presenting with acute cholangitis, managed with percutaneous transhepatic cholangiography (PTC) and endoscopic retrograde cholangiopancreatography (ERCP). Case II illustrates a similar presentation complicated by myocardial infarction, necessitating urgent biliary decompression via PTC and subsequent unsuccessful endoscopic stenting. Case III highlights the diagnostic difficulty in a stable patient with inconclusive diagnostic imaging. This case series emphasizes the importance of considering sump syndrome diagnosis in patients with a history of CDD or open cholecystectomy, especially in elderly patients and those who come from regions where alternative biliary interventions may be limited.

## Graphical abstract

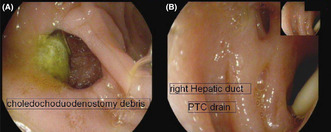

## 1 INTRODUCTION

Sump syndrome, a rare and often underreported medical condition, occurs as a long‐term complication in patients with a history of side‐to‐side choledochoduodenostomy (CDD). The aforementioned surgical procedure was once considered a last resort in managing biliary duct obstructions, which involves creating an anastomosis between the common bile duct (CBD) and the duodenum.^1^ The term “sump” refers to the remaining portion of the CBD between the Billary‐duodenal anastomosis and the ampulla of Vater. Moreover, this segment acts as a stagnant reservoir prone to accumulating debris, stones, and static bile, leading to bacterial proliferation. This, in turn, predisposes individuals to a high risk of recurrent cholangitis, pancreatitis, and, in rare cases, hepatic abscess if left untreated.^2^

In the modern era, this kind of clinical entity can be easily missed due to the wide replacement of CDD with endoscopic retrograde cholangiopancreatography (ERCP). However, we still face its consequences in the elderly population and in individuals of global migration from developing countries where CDD is the mainstay approach for biliary obstruction.^3^ Therefore, this study aimed to contribute to this relatively under‐researched field by conducting a case series of sump syndrome.

## 2 CASE PRESENTATION

### 2.1 Case history and examination

#### 2.1.1 Case I

A 35‐year‐old Filipino woman presented to the emergency department (ED) with right upper quadrant abdominal pain. She also reported nausea, vomiting, and yellowish skin discoloration. The patient had a previous history of open cholecystectomy done in Filipin 2 years before presentation. The patient was not able to provide accurate details about her surgery. On examination, she was febrile with a temperature of 38.7 C, tachycardic with a heart rate (HR) of 127 beats per minute, and hypotensive with blood pressure (BP) of 88/55 mmHg. Abdominal examination revealed diffuse tenderness all over the abdomen without guarding or rigidity.

#### 2.1.2 Case II

The second case is a 52‐year‐old Filipino woman with a past medical history of hypertension and open cholecystectomy, which was done at the age of 30 years in Filipin. She presented to the ED with a complaint of epigastric pain and vomiting. On examination, the temperature was 37.8 C with an HR of 110 beats per minute and BP of 77/48.

#### 2.1.3 Case III

A 65‐year‐old Pakistani man with a past medical history significant for diabetes mellitus, hypertension, coronary artery disease, and a remote history of cholecystectomy. He had recurrent admissions and visits to the ED because of upper abdominal pain and nausea. The patient was vitally stable, with only mild tenderness in the epigastric area.

### 2.2 Diagnosis, investigation, and treatment

#### 2.2.1 Case I

Blood laboratory testing showed high inflammatory markers and high liver function tests (LFT) with a cholestatic picture. She was initially admitted to the medical intensive care unit (MICU), resuscitated with intravenous fluids and vasopressors, and was started on broad‐spectrum antibiotics. Ultrasound (US) of the abdomen showed hepatic pneumobilia with a normal CBD diameter. Magnetic resonance imaging (MRI)/magnetic resonance cholangiopancreatography (MRCP) revealed features of acute ascending cholangitis with a stricture at hilar confluence, markedly dilated intrahepatic bile ducts, and pneumobilia without stones. After discussion between the gastrointestinal (GI) and hepato‐pancreato‐biliary (HPB) teams, a decision to go first for percutaneous transhepatic cholangiography (PTC) by interventional radiology (IR) was made as the patient was hemodynamically unstable.

#### 2.2.2 Case II

Labs showed markedly high inflammatory markers, and LFT showed a rise in liver enzymes with predominant direct hyperbilirubinemia. She was admitted under MICU care, resuscitated with IV fluids and vasopressors, and received broad‐spectrum antibiotics. US of the abdomen showed dilated CBD of 7.7 mm with two stones, the largest being 1.6 cm, and minimal pneumobilia. A few hours after the presentation, the patient developed one chest pain with high troponin and transient ischemic electrocardiogram (ECG) changes. She was labeled as type II myocardial infarction (MI) and started on dual anti‐platelet treatment. She was deemed unstable for endoscopy, and PTC was done for urgent biliary decompression.

#### 2.2.3 Case III

His labs were unremarkable, as was his computed tomography (CT) of the abdomen, done to rule out bowel obstruction. US of the abdomen was suggestive of intrahepatic pneumobilia. Endoscopic examination showed only anatomical evidence of CDD. The patient was diagnosed with sump syndrome and was planned for ERCP as an outpatient as his abdominal pain improved during the admission.

### 2.3 Outcome and follow up

#### 2.3.1 Case I

**FIGURE 1:**
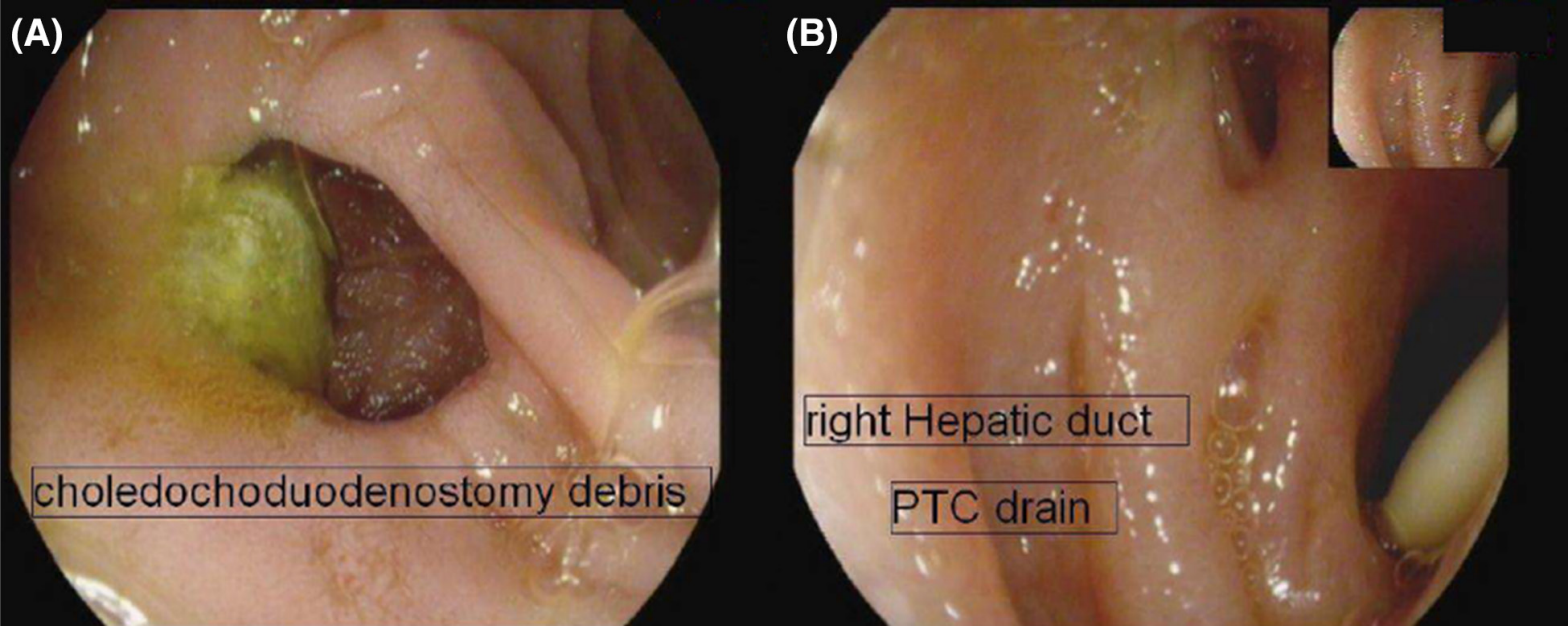
Forward endoscopic view of the first patient demonstrating: (A) choledochoduodenostomy (CDD) with debris visualized. (B) Right hepatic duct with percutaneous transhepatic cholangiography (PTC) drain in place.

After stabilization, forward‐viewing endoscopy showed CDD in the first part of the duodenum, with non‐occluding food debris, which was removed (Figure 1). No strictures were seen during the endoscopy. Biliary sphincterotomy and balloon sweep were done through ERCP. Later, the patient recovered and was discharged after a 30‐day hospital stay.

#### 2.3.2 Case II

Once stabilized, cholangiogram showed a choledocho‐duodenal fistula in the first part of the duodenum, later confirmed as CDD. Biliary sphincterotomy and balloon sweep were done, and no stones or debris were seen. A CBD stent was placed; however, it was coiling and projecting out of the surgical anastomosis site and was removed by endoscopy in the same setting. Before discharge, myocardial perfusion imaging showed no fixed or reversible perfusion abnormalties, She was labeled as type II MI and kept on aspirin alone. The patient was assessed clinically, showed improvement and later discharged home.

#### 2.3.3 Case III

The patient was stable clinically and lab‐wise. However, he did not show up for the ERCP appointment. Table 1 demonstrates the clinical and diagnostic characteristics of the three patients.

**TABLE 1:** Clinical and diagnostic characteristics of the three patients.

	Patient I	Patient II	Patient III
Age (years)	35	52	65
Gender	Female	Female	Male
Comorbidities	None	HTN	DM, HTN, CAD
Surgical history	Open cholecystectomy	Open cholecystectomy	Open cholecystectomy
Time interval between surgery and appearance of symptoms	Two years	More than 20 years	15 years
Presentation	Acute RUQ pain and vomiting with sepsis	Acute epigastric pain and vomiting with sepsis	Recurrent epigastric pain and nausea
Vitals on presentation	Unstable	Unstable	Stable
AST (U/L)	37	292	20
ALT (U/L)	56	348	23
ALP (U/L)	171	154	173
Total bilirubin (umol/L)	31	45	9.8
Blood Cultures	Escherichia coli	Klebsiella pneumoniae	None
Management	Stabilization + IV antibiotics + PTC + biliary sphincterotomy and balloon sweep	Stabilization + IV antibiotics + PTC + biliary sphincterotomy and balloon sweep	Symptomatic management, ERCP was planned, but the patient did not show up

Abbreviations: CAD, coronary artery disease; DM, diabetes mellitus; HTN, hypertension; PTC, percutaneous transhepatic cholangiography; RUQ, right upper quadrant.

## 3 DISCUSSION

Sump syndrome is an uncommon, long‐term complication seen in patients with a history of a side‐to‐side CDD, a standard surgical procedure to manage biliary tract disease in the pre‐ERCP era. CDD is considered the simplest form of bilioenteric anastomosis, with only minimal alteration to the normal anatomy. In the setting of a side‐to‐side CDD, decompression of the biliary duct is achieved through an anastomosis created between the CBD and the duodenum. The CBD segment distal to the anastomosis consequently transforms into a poorly drained reservoir prone to accumulating debris, stones, and static bile, hence the term “sump.”

The prevalence of sump syndrome following side‐to‐side CDD is uncertain and has been reported to vary widely between 0% and 15.7%.^4^, ^5^ This variation in prevalence may be explained by the lack of a precise definition of sump syndrome in the literature. However, most definitions are consistent with obstruction of the distal CBD by food, stones, bile, or debris in patients with bilioenteric anastomosis, leading to biliary/pancreatic complications.^6^ Although sump syndrome has been related to bilioenteric anastomosis in general, it is usually observed following surgical CDD procedures. Less commonly, sump syndrome has been reported following hepaticojejunostomy (HJ) procedures.^7^ Schreuder A. et al. conducted a matched case–control study, comparing outcomes of CDD with HJ, and found a 7.7% and 0% prevalence of sump syndrome in these groups, respectively.^6^ Sump syndrome has also been reported following a liver transplant with HJ^8^ and Endoscopic ultrasound (EUS)‐guided CDD.^9^ All of our three patients developed sump syndrome following CDD.

Overall, sump syndrome is rarely seen nowadays due to the advancement of EUS, ERCP, and laparoscopic cholecystectomy, with less side‐to‐side CDD being performed. Despite that, sump syndrome is still seen in elderly patients with a remote history of CDD or in patients from countries where ERCP may not always be feasible. The clinical presentation is non‐specific, with epigastric and right upper quadrant pain being reported the most. Other presenting symptoms include fever, chills, and jaundice. Presentation often correlates with cholangitis, pancreatitis, or liver abscess.^1^, ^10^, ^11^ Similarly, laboratory findings are not specific and include raised inflammatory markers, liver enzymes, and positive blood culture. Because of the non‐specific presentation and its rarity, diagnosis of sump syndrome can be challenging and often be delayed, affecting patient care. Furthermore, the long interval between the surgery and the emergence of symptoms may pose an additional challenge in diagnosing sump syndrome, as previous medical records may not be available, and patients may not be fully aware of the surgery details. A retrospective French study showed a five‐year median time interval between surgery and the onset of symptoms.^10^ In our case series, all three patients presented with epigastric pain and jaundice, and two of them required admission to the MICU. The time of presentation ranged between two and thirty years.

Diagnostic imaging is critical in diagnosing sump syndrome in most cases. US, CT, and MRI/MRCP are often helpful. Radiologic findings suggestive of sump syndrome include pneumobilia, biliary duct dilatation, and the presence of debris or stones in the distal CBD.^12^, ^13^ In addition, radiologic findings of complications of sump syndrome, including cholangitis, liver abscess, and pancreatitis, may be present. It is worth mentioning that pneumobilia, defined as air in the biliary tree, may reflect a previous bilioenteric anastomosis surgery but does not confirm the presence of sump syndrome per se. Other causes of pneumobilia include post‐sphincterotomy, biliary cancer, and bilioenteric fistula.^3^ ERCP or PTC has been suggested to be necessary for confirming the diagnosis of sump syndrome.^12^ The first two patients in our study were diagnosed via imaging and confirmed with direct endoscopy. However, the third patient's imaging was inconclusive, and the diagnosis was only made after endoscopic visualization. Other imaging that can be helpful in the diagnosis include upper GI fluoroscopic studies, which may demonstrate reflux of contrast into the biliary tree through a patent anastomosis of the CDD outlining the CBD and intrahepatic biliary tree.^12^ In addition, Sait S. et al. reported a sump syndrome case diagnosed via microvascular flow imaging (MVI).^14^

Sump syndrome management aims to improve the biliary drainage at the distal CBD segment and address any underlying infection. There are no current guidelines to guide the treatment of sump syndrome, and its treatment is tailored to each patient based on many factors, including severity of symptoms, presence of complications, anatomical modifications, recurrence, and patient preference. Management reported in the literature ranges from a “wait‐and‐watch” approach^1^ to surgical treatment by conversion to another biliary diversion.^11^ Most cases, however, are treated with endoscopic sphincterotomy and duct clearance to facilitate the drain through the ampulla of Vater, with or without dilatation of the anastomosis to facilitate the drainage through it. Specific endoscopic techniques to treat sump syndrome have also been reported, including closure of CDD using an over‐the‐scope clip^15^ or a cardiac septal occluder device.^16^ Granata A. et al. also reported a sump syndrome case treated with CDD revision using the OverStitch device.^17^ The first and second patients in our study were treated with PTC to decompress the biliary tract and IV antibiotics, as they were hemodynamically unstable. Once stable, endoscopic sphincterotomy and balloon sweeps were done for both patients. The third patient, however, was planned for ERCP with possible biliary sphincterotomy as an outpatient, but he did not show up to the appointment.

## 4 CONCLUSION

Sump syndrome is an uncommon late complication of CDD. It is important to consider the diagnosis in patients with a history of open cholecystectomy, particularly in those who migrate from developing countries. The presentation may range from acute severe, mimicking acute ascending cholangitis, to chronic recurrent abdominal pain without evidence of inflammation. Management is a case‐by‐case decision, with principal management aims to decompress the biliary tract and address any underlying sepsis.

## AUTHOR CONTRIBUTIONS

**Shahem Abbarh:** Conceptualization, Formal analysis, Methodology, Validation, Visualization, Writing – original draft, Writing – review & editing. **Bisher Sawaf:** Conceptualization, Formal analysis, Methodology, Validation, Visualization, Writing – original draft, Writing – review & editing. **Hussam Almasri:** Conceptualization, Investigation, Methodology, Validation, Visualization, Writing – original draft, Writing – review & editing. **Girisha Balaraju:** Conceptualization, Validation, Visualization, Writing – original draft, Writing – review & editing. **Mhd Kutaiba Albuni:** Conceptualization, Methodology, Visualization, Writing – original draft, Writing – review & editing. **Shaher Abbarah:**Conceptualization, Data curation, Supervision, Validation, Visualization, Writing – original draft, Writing – review & editin. **Ashraf I. Ahmed:** Conceptualization, Data curation, Validation, Visualization, Writing – original draft, Writing – review & editin. **Abdellatif Ismail:** Conceptualization Validation Visualization, Writing – original draft, Writing – review & editing. **Saad Rashid Mohammad Al Kaabi:** Conceptualization, Data curation, Supervision, Validation, Visualization, Writing – original draft, Writing – review & editing.

## FUNDING INFORMATION

This research did not receive any specific grant from fund‐ing agencies in the public, commercial, or not‐ for‐profit sectors.

## CONFLICT OF INTEREST STATEMENT

The authors report no conflict of interest.

## ETHICS STATEMENT

The article was approved by the Institution Review Board at Hamad Medical Corporation.

## CONSENT

A written informed consent was obtained from the patients for the publication of all images, clinical data and other data included in the manuscript. All identifying information has been removed.

## Data Availability

The data that support the findings of this study are available from the corresponding author upon reasonable request.
